# Discovering collectively informative descriptors from high-throughput experiments

**DOI:** 10.1186/1471-2105-10-431

**Published:** 2009-12-18

**Authors:** Clark D Jeffries, William O Ward, Diana O Perkins, Fred A Wright

**Affiliations:** 1 Eshelman School of Pharmacy, University of North Carolina at Chapel Hill, NC, USA; 2 Renaissance Computing Institute, University of North Carolina at Chapel Hill, NC, USA; 3 NHEERL Environmental Carcinogenesis Division, United States Environmental Protection Agency, Research Triangle Park, NC, USA; 4 Department of Psychiatry, University of North Carolina at Chapel Hill, NC, USA; 5 Department of Biostatistics, University of North Carolina at Chapel Hill, NC, USA

## Abstract

**Background:**

Improvements in high-throughput technology and its increasing use have led to the generation of many highly complex datasets that often address similar biological questions. Combining information from these studies can increase the reliability and generalizability of results and also yield new insights that guide future research.

**Results:**

This paper describes a novel algorithm called BLANKET for symmetric analysis of two experiments that assess informativeness of descriptors. The experiments are required to be related only in that their descriptor sets intersect substantially and their definitions of case and control are consistent. From resulting lists of n descriptors ranked by informativeness, BLANKET determines **shortlists **of descriptors from each experiment, generally of different lengths p and q. For any pair of shortlists, four numbers are evident: the number of descriptors appearing in both shortlists, in exactly one shortlist, or in neither shortlist. From the associated contingency table, BLANKET computes Right Fisher Exact Test (RFET) values used as scores over a plane of possible pairs of shortlist lengths [1,2]. BLANKET then chooses a pair or pairs with RFET score less than a threshold; the threshold depends upon n and shortlist length limits and represents a quality of intersection achieved by less than 5% of random lists.

**Conclusions:**

Researchers seek within a universe of descriptors some minimal subset that collectively and efficiently predicts experimental outcomes. Ideally, any smaller subset should be insufficient for reliable prediction and any larger subset should have little additional accuracy. As a method, BLANKET is easy to conceptualize and presents only moderate computational complexity. Many existing databases could be mined using BLANKET to suggest optimal sets of predictive descriptors.

## Background

In contemporary high-throughput experiments, very many descriptor values can be measured, leading to the issue of correction for multiple testing to minimize false positives at the cost of a high number of false negatives. Reconciliation entails compromises that are to some extent arbitrary. A deterministic method is needed for selecting a minimal, distinguished set of descriptors that collectively provide effective, efficient prediction. Researchers can subsequently investigate members of such a subset to determine exactly how they are related (e.g. are they genetically or chemically related?) and perhaps why they should be inherently associated with predictions (e.g. are some members of the shortlists components of a certain biochemical pathway?).

Meta-analysis is the general body of knowledge that addresses integrating results from multiple experimental programs on one topic; the purpose of this paper is to suggest inclusion of BLANKET as an additional technique [3]. Regarding related papers, we note that Hess and Iyer found that Fisher's combined p method applied to microarray data from spike-in experiments with RT-qPCR validation usually compared favorably to other methods [4]. However, they observed that other probe level testing methods generally selected many of the same genes as differentially expressed. So the method of finding differentially expressed genes is not the critical issue. As they further noted, current methods for analyzing microarray data do better at ranking genes rather than maintaining stated false positive rates.

Lists of descriptors ranked by informativeness are often encountered in the general pursuit of relationships among diseases, physiological processes, and the action of small molecule therapeutics. Notable examples include the Connectivity Map by Lamb et al. and the generation of quantitative structure-activity relationships (QSAR) [5-7]. Kazius et al. considered N compounds, each of which either is or is not toxic (e.g. mutagenic) [8]. They characterized compounds by substructures, each compound either including or not including a given substructure. Inclusion of any substructure thereby can be considered as a potential toxicity descriptor, and the point of Kazius et al. was analysis of single experiments to determine toxicity. BLANKET could be applied to the outcomes of two experiments that use the same set of descriptors.

Regarding genes as descriptors (that is, expression of mRNAs or proteins), a vast, public repository of data that should support discovery of distinguished descriptor lists is supported by the Gene Expression Omnibus (GEO) project. GEO predominantly stores gene expression data generated by microarray technology [9-11]. Another huge data resource is Oncomine as developed by Rhodes et al. [12,13]. Oncomine includes statistical reports on some 18,000 cancer gene expression microarrays.

## Methods

**Figure 1:**
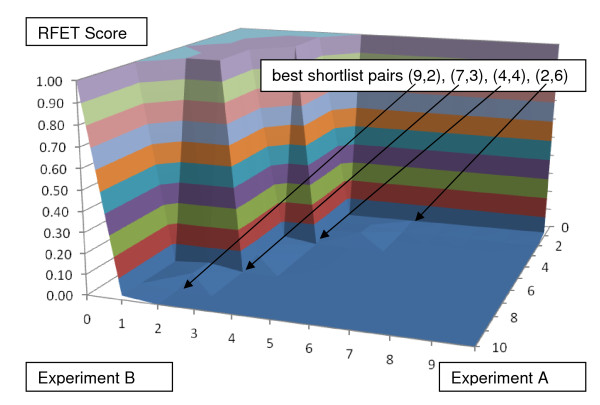
**BLANKET applied to comparison of two illustrative lists of 500 descriptors**. The first ten from Experiment A appear in scrambled order within the first ten of Experiment B. BLANKET suggests that four combinations of shortlists are sufficiently coincidental to meet a p-value of .05. Note that three of the four selected shortlist pairs (p, q) have unequal numbers of selected descriptors.

Presented first are two synthetic examples. Suppose the number of distilled descriptors n = 500 and the ranked list for Experiment A is simply labeled 1, 2, ..., 500. Suppose in the ranked list for Experiment B, the first ten are a random permutation of 1, 2, ..., 10, and the other 490 are a random permutation of 11, 12,...,500. We would expect BLANKET to suggest an optimal subset of the first ten just as is shown in Figure 1. (Should the very first descriptor from one experiment be also the first of the other, then BLANKET simply declares that descriptor to be the optimal subset.) Note the appearance of the BLANKET surface: a plateau of RFET values near 1 for very low p+q abruptly falls to a floor of values near 0 as p+q increases. By definition of RFET, the extreme pairs with p = 0 or n, or q = 0 or n values have RFET = 1, a property of all BLANKETs. So to speak, the square BLANKET surface is supported at value 1 around its edge and dips to positive values ≤1 in its interior. Random BLANKETs (from randomly sorted lists) seem to have no such patterns of plateaus and floors and they generally have larger minimum values.

**Figure 2:**
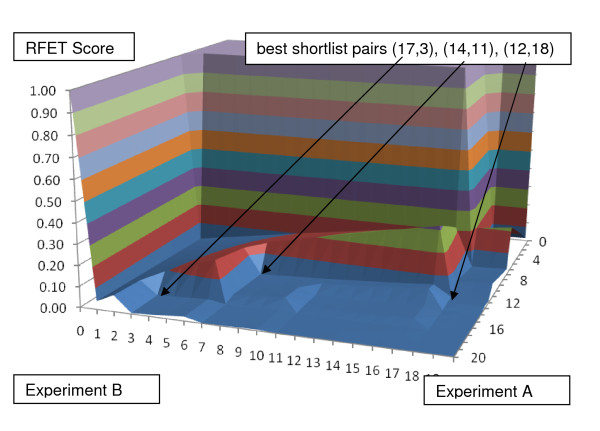
**BLANKET applied to a second set illustrative lists of n = 500 descriptors**. Descriptors in Experiment A are ranked in canonical order 1, 2, ..., 500. To the same ranking, weighted noise is added to arrive at an Experiment B ranking of 7, 26, 17, 32, 21, 34, 12, 46, 49, 14, 57, 54, 67, 19, 61, 28, 1, 15, 82,... BLANKET finds no shortlists of length at most 10 that meet the criteria for significance (p-value ≤ .05, corresponding to RFET < .00105). However, BLANKET finds three shortlist pairs as shown of length < 20 that do meet the same (RFET < .00800). Thus BLANKET, not knowing the effects of noise, would recommend to the researcher these descriptors for further investigation. Note the characteristic sharp decline in RFET values near the chosen shortlists. This example is relevant to the case of one experiment performed with great accuracy and the other with substantial noise. Note that each selected shortlist pair (p, q) has unequal numbers of descriptors selected from both experiments (p ≠ q).

As a second synthetic example, suppose Experiment A descriptors again have canonical ordering 1,2,...,500 while Experiment B has the same with local permutations from weighted noise. Figure 2 shows that the noise in the ranked lists can be sufficient to preclude shortlists of length < 10, but three survive the < 20 criterion. Again there is a plateau of RFET values near 1, falling abruptly to a floor of near 0 values.

Next the BLANKET method will be used to evaluate data from a classic microRNA (miRNA) microarray paper by He et al. [14]. The spreadsheet data from the paper are in the NCBI/NLM/GEO web site with Accession number GSE2399, entitled "MicroRNA expression in lymphoma lines" [9]. Two experiments evaluated miRNA expression levels in cell lines OCI-Ly4 and OCI-Ly7 (both relative to the same control cells); these cell lines carry amplification of genomic region of interest 13q31-q32 that is thought to be oncogenic. In each experiment the results from the cell lines were compared to the same measurements of normal B-cells.

He et al. measured in quadruplicate for cases and for controls 190 mature miRNA levels for normal B-cells and several cell lines including OCI-Ly4 and OCI-Ly7. Thus Experiment A includes the 190-by-8 output matrix of Normal (control) B-cell miRNA values versus OCI-Ly4 (case) miRNA values, and as Experiment B the same from OCI-Ly7 values. This yields p-values and hence rankings of the two lists of 190 miRNAs.

We tested the hypothesis that the same miRNAs can differentiate control B-cells from both of the two cases OCI-Ly4 and OCI-Ly7 by attempting to find informative subsets of the 190 probes.

**Figure 3:**
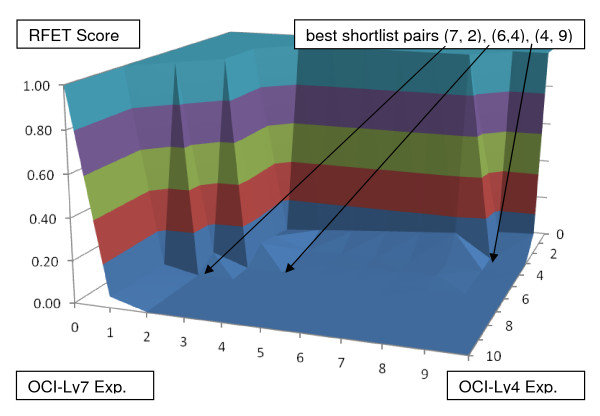
**BLANKET applied to ranked list date of 190 descriptors from He et al. (He et al. 2005)**. BLANKET suggests that three combinations of shortlists are sufficiently coincidental to meet a p-value of .05. Note that each selected shortlist pair (p, q) has unequal numbers of descriptors selected from both experiments (p ≠ q).

The BLANKET for these data is shown as the surface in Figure 3. This BLANKET finds that three combinations of shortlists that achieve the p, q ≤ 10 threshold for n = 200, namely, 1.50E-03 (Table 1). That is, RFET = 1.17E-03 for (7,2); RFET = 7.02E-05 for (6,4); and RFET = 2.91E-04 for (4,9). The top 7 of the OCI-Ly4 list are: let-7e, -7g, -7c, -7f, -7d, -7a, and miR-373*. The top 9 of the OCI-Ly7 list are miR-373*, let-7a, -7c, -7f, miR-138, -423, -15a, -223, and let-7g.

**Table 1:** BLANKET multiple comparison-corrected significance threshold values for p-value 0.05.

n	p, q ≤ 20	p, q ≤ 10
100	0.00424	0.00192
200	0.00549	0.00150
300	0.00666	0.00119
400	0.00657	0.00104
500	0.00800	0.00105

It is already obvious from the heatmap in Figure 1 of the He paper that the let-7 family is distinguished by case versus control. Aside from the let-7 family, the union of the BLANKET shortlists contains five other miRNAs: hsa-miR-373*, -138, -423, -15a, and -223. There is an interesting alignment among these:

hsa-miR-138   5' AGCU-GGUGUUGUGAAUCAGGCCG 3'

                 |||| |||

hsa-miR-423   5' AGCUCGGUCUGAGGCCCCUCAGU 3'

This alignment invites investigation because the bases near the 5' terminus (the "seed region") are generally thought by miRNA researchers to be most important in terms of targeting and gene regulation [15]. Possibly the similarity of miR-138 and miR-423 in this respect implies the two are actually redundant; redundancy is considered a hallmark of miRNA targeting efficacy [16]. Redundancy might allow fine tuning when one is upregulated in case and the other downregulated, as is so for these miRNAs. Otherwise, shortlisted descriptors might exhibit consistent change associations between the two experiments, as is the case for 7 of the other 9 miRNAs in the BLANKET union of shortlists for these data.

BLANKET is next applied to suggest shortlists of genes from experiments with lung adenocarcinoma measurements versus control tissue measurements in microarray studies by Stearman et al. and Bhattacharjee et al. [17,18]. Each study contains a statistical contrast of normal lung tissue versus adenocarcinoma tissue. The gene symbols and associated p-values from each study can be downloaded from Oncomine. The Stearman study considers 7815 genes (excluding ESTs and multiple measurements for one gene); the number for Bhattacharjee is 7160.

**Figure 4:**
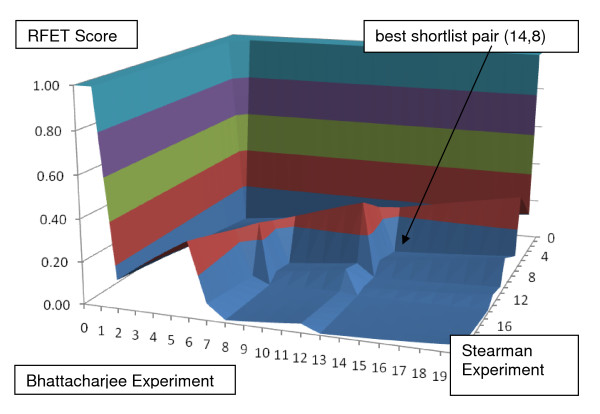
**BLANKET applied to comparison of 289 genes within lung cancer microarray studies of Stearman and Bhattacharjee**. One pair of shortlists with 14 descriptors from the first and eight from the second yields a RFET score = .0044; this is less than .0066, the level that insures a p-value significance level (.05) for any shortlists with twenty or fewer members from a universe of 300 members.

Ranked by lowest p-values, the top 1000 genes in each experiment can be selected. The intersection of the lists can be reranked to a list of 289 genes that are possibly informative in both experiments. BLANKET yields the surface in Figure 4.

From the Stearman data BLANKET chooses 14 genes: GPC3, SOX4, GRK5, ADH1B, CLEC3B, MFAP4, TEK, FH1, AOC3, TBX2, COX7A1, TGFBR3, MYLK, VWF. BLANKET chooses 8 genes from the Bhattacharjee data: HYAL2, GRK5, SPOCK2, ENO1, SEMA5A, CDH5, VWF, COX7A1. Thus the intersection is {GRK5, COX7A1, VWF} with RFET = 4.42E-03.

Interestingly, several additional papers connect some of the shortlisted genes with lung cancer. Regarding GPC3, Powell et al. used microarrays to identify GPC3 as one of several genes the expression of which was lower in the healthy lung tissue of smokers than in nonsmokers and was lower in tumor tissue than in healthy tissue [19]. Additionally, northern blot analysis demonstrated that GPC3 expression was absent in 9 of 10 lung cancer cell lines. Regarding ADH1B, Kopantzev et al. employed cDNAs sequencing and RT-qPCR analysis to measure genes differentiated in comparison of human fetal versus adult lungs and in normal lung tissue versus non-small lung cell carcinomas [20]. ADH1B was one of 12 genes found to have opposite differentiation in the two comparisons. Regarding CLEC3B, reduced plasma levels have long been associated with cancer and metastasis [21]. Regarding TEK, Millauer et al. implicated TEK among growth factor receptor tyrosine kinases in angiogenesis, and Findley et al. demonstrated that VEGF regulates TEK signaling [22,23]. Regarding AOC3, Singh et al. found that expression may contribute to the functional heterogeneity of endothelial cells within the lung to create distinct sites for the recruitment of inflammatory cells [24]. Regarding HYAL2, Li et al. studied genetic aberrations in the genes HYAL2, FHIT, and other genes in paired tumors and sputum samples from 38 patients with stage I non-small cell lung cancer and in sputum samples from 36 cancer-free smokers and 28 healthy nonsmokers [25]. They found HYAL2 and FHIT were deleted in 84% and 79% tumors and in 45% and 40% paired sputum samples. Regarding ENO1, Chang et al. observed that only a limited number of immunogenic tumor-associated antigens have been identified and associated with lung cancer [26]. They reported up-regulation of ENO1 expression in effusion tumor cells from 11 of 17 patients compared with human normal lung primary epithelial and non-cancer-associated effusion cells. Regarding MYLK, Soung et al. analyzed exons 6 and 7 encoding the kinase domain for somatic mutations in 60 gastric, 104 colorectal, 79 non-small cell lung, and 54 breast cancers [27]. They found one MYLK2 mutation in lung adenocarcinomas, but not in other cancers. Regarding SEMA5A, Sadanandam et al. demonstrated an association between the expression of SEMA5A and Plexin B3 and the aggressiveness of pancreatic and prostate cancer cells [28]. They deduced that SEMA5A is among functional tumor-specific CAM genes, which may be critical for organ-specific metastasis. Regarding CDH5 and intersection gene VWF, Smirnov et al. reported increased numbers of endothelial cells in peripheral blood of cancer patients [29]. They found expression of VWF, DTR, CDH5, TIE, and IGFBP7 genes discriminated between cancer patients and healthy donors with a receiver operating characteristic curve accuracy of 0.93. Of the other two genes in the intersection, GRK5 is a G protein-coupled receptor kinase and is highly expressed in lung [30]. Lastly, COX7A1 is 13 kb from and possibly co-expressed with FXYD5 (alias dysadherin), a cancer-associated cell membrane glycoprotein that promotes experimental cancer metastasis [31].

In summary, there are potential lung cancer connections with genes in the Stearman-Bhattacharjee BLANKET shortlists. This illustrates the main output of BLANKET, namely, suggestions to researchers of small subsets of genes especially worthy of further investigation.

**Figure 5:**
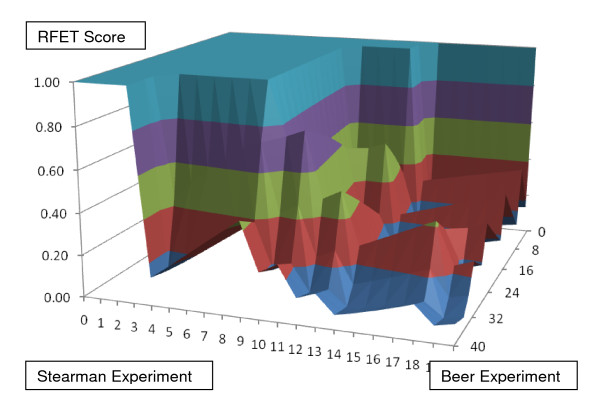
**BLANKET applied to comparison of lung cancer microarray studies of Beer and Stearman**. The method fails to find a threshold pair with low RFET score < .00800, which would be sufficient for shortlists with up to 20 members to have statistical significance in a universe of 489 descriptors. This surface is more organized than random BLANKETs, since there is a sharp decrease from 1 to low values, but it is less organized than those in Figures 3 and 4.

The next analysis pertains to an instance in which BLANKET does not suggest informative shortlists; this example compares results of Stearman with another lung cancer study by Beer et al. [32]. Preprocessing starting with the 1000 most differentiated gene lists leads to selection of 489 shared genes. As shown in Figure 5, the BLANKET surface does not display a sharply defined subset of informative descriptors, that is, no plateau that falls precipitously to a floor of RFET values near zero.

Lastly, BLANKET applied to the third possible combination of tests (Beer-Bhattacharjee) is not interesting. It finds a very small subset pair (2,5). Such small shortlists are evident by inspection because the top two for Beer are ADH1B and CLEC3B while the top five for Bhattacharjee are GPC3, SOX4, GRK5, ADH1B, CLEC3B.

## Implementation

Following is a pipeline (for R code see Additional File 1) for processing descriptors measured in two experiments called A and B. Each experiment routinely yields a matrix of values with descriptors labeling the rows and samples labeling the columns. An additional column is the informativeness of each descriptor from application of Student t-testing or another method; that additional column is used to rank the descriptors by informativeness, yielding the two ranked descriptor lists used as inputs by BLANKET.

Considerable preprocessing might be needed to derive the ranked lists. This is because the raw data (e.g. mRNA microarrays) typically have many thousands of descriptors, from which one distills hundreds that are significantly up or down in case versus control; the researcher might wish to treat up- and downregulated genes separately. The intersection of the two lists must be found and then a selection made of the topmost descriptors (such as the top 500) of the two lists. Some of the top 500 in one list might not be in the top 500 of the other, so a second intersection is needed to yield a list of genes with different rankings in the two experiments, that is, somewhat fewer, shared, ranked descriptors suitable for BLANKET. Real data tested in preparation of this paper yielded an intersection n = ~250 to ~450 descriptors.

Using the list of shared descriptors and selecting the top p descriptors from A and the top q descriptors from B yields a contingency table. Over the discrete plane of all possible pairs, RFET values can be represented as a blanket-like surface.

Table 1 shows for various n values and two reasonable upper limits on p and q the low RFET values that are attained by only 5% of random lists. For n = 100, 200, and 300, the entries are based on 500 simulations. For n = 400 and 500, they are based on 1000 simulations. Thus, for example, a researcher who distills experimental information down to two ranked lists of 200 descriptors and finds a shortlist pair (p, q) with p and q ≤ 20 and RFET = .004 (< .00549) can dismiss the null hypothesis with a 5% false positive rate.

After finding shortlist pairs that provide RFET values lower than the values in Table 1, the researcher should select shortlists as follows: For each selected shortlist pair (p, q), no other shortlist pair (p', q') also has p' ≤ p, q' ≤ q, and p'+q' < p+q.

All such values and corresponding descriptors should be noted by the researcher. All genes that achieve a level of informativeness discovered in a BLANKET selection might be considered. That is, the union of all the descriptors in the shortlists might be informative, as well as, of course, the intersection. If several pairs of shortlists fulfill this condition, then minimizing the RFET values or minimizing p+q might yield especially interesting shortlist pairs.

## Results

If two experiments of case versus control have substantially overlapping descriptor sets and a consistent, binary categorization of outcome, then standard statistical analyses can provide two lists, ranked by informativeness, of the shared descriptors. The ranked lists suggest two questions:

Question 1: From the results of the two experiments, is there a minimal subset of descriptors that predicts experimental outcome much better than smaller subsets and about as well as any larger subsets?

Question 2: If existence of such a minimal subset is indicated, then what are its members?

The focus here is on one method that answers these questions. We call our method BLANKET; this is not an acronym, but merely a term suggestive of a blanket-like surface suspended above a plane of shortlist length pairs.

Suppose two experiments such as microarray analyses investigate informativeness of descriptors relative to a property of samples. Here a descriptor (predictor) is any tested type of measurement, such as detection of messenger RNA of a certain gene in a microarray experiment (a continuous variable) or presence or absence of a certain chemical substructure in a compound evaluated for toxicity (a binary variable). A property of the samples could be case versus control, survival time, or another characteristic or outcome of interest. BLANKET treats the set of shared descriptors as two ranked lists.

The informativeness of each descriptor, considered in isolation, can be determined by a by t-test, z-test, or other method; our only requirement is that informativeness analysis for each experiment yields a ranked list. The two experiments might use the same or different definitions of informativeness.

The basic idea of BLANKET is consideration of **shortlists **of descriptors from each experiment, say the top p of n descriptors of Experiment A and the top q of the same n descriptors of Experiment B. For any such pair of shortlists, four numbers are evident: the number of descriptors appearing in both shortlists, in exactly one shortlist, or in neither shortlist. The sum of all four is n; the sum of the first two is p; and the sum of the first and third is q. BLANKET computes Right Fisher Exact Test (RFET) values used as scores over a discrete plane of all possible pairs of shortlist lengths (so all (p, q) with 0 ≤ p, q ≤ n). BLANKET then chooses one pair or a few pairs with RFET score less than a threshold; the threshold depends upon n and upper bounds of shortlist lengths. The threshold has been determined by simulations and represents a quality of RFET value achieved by only 5% of random lists. A further property of a pair (p, q) selected by BLANKET is parsimony, that is, that no other pair (p', q') exists with p' ≤ p, q' ≤ q, p'+q' < p+q, and an RFET score that also survives the threshold. Multiple shortlists could be scored by smallness of p+q.

Furthermore, we seek to represent the information to the researcher in a visual form such as an Excel spreadsheet surface graph that invites assessment based upon a researcher's experience with data of a given type, much in the manner of the commonplace heatmap.

## Theoretical basis

Our approach is to consider the RFET value for all combinations of shortlist lengths 10 or 20 within ranked descriptor lists of length n = 100, 200, 300, 400, or 500. In the grid of lengths, this can be thought of as the examination of all p-by-q rectangles of RFET values within a given nxn square, subject to p ≤ n and q ≤ n. The RFET attaining the minimum nominal p-value is then compared to the null distribution of such minimum p-values, obtained via permutation, which assumes that the orderings of the two lists of descriptors are random. The corresponding 0.05 quantile values are used as rejection thresholds for controlling the overall Type I error at 0.05.

Formally, the approach is the single-step Westfall-Young permutation p-value for potentially correlated tests, which controls the family-wise error and avoids the excessive conservativeness of Bonferroni bounds [33]. Furthermore, the approach has an exact interpretation as a kind of randomization test of a statistic (minimum nominal p-value) in a population of equally likely outcomes (alignment of descriptor lists) conditioned on some aspect of the data (descriptor identities) [34]. This is an attractive approach, as it makes very few assumptions about the data and is entirely nonparametric.

## Discussion

The term BLANKET reflects the shapes of the surfaces in Figures 1, 2, 3, 4 and 5. We can reason as follows about the shape. If the threshold for at least one of the lists is too strict (very small or zero) so that one shortlist is empty or small and there is no intersection, then RFET = 1; likewise, if at least one shortlist is the universe of descriptors, then RFET = 1. Thus the boundary of the BLANKET surface over the full range of all threshold pairs necessarily has fixed value 1. This insures that seeking interior points with relatively low RFET values on the surface makes sense.

To our knowledge, BLANKET is a novel means for nominating distinguished subsets of descriptors from data from two experiments. BLANKET suggests shortlists (subsets) of genes from each list, where the shortlists achieve a certain level of informativeness individually. The subsets then collectively differentiate case from control. While the preprocessing considers the full ranked lists, BLANKET does not make global declarations. That is, BLANKET ignores very uninformative descriptors but can tolerate descriptors with marginal p-values provided they consistently appear among the best found of ranked lists.

Other related scores that might be substituted for the RFET score are Pearson's chi-square test and the G-test [35,36]. Once a distinguished set of descriptors has been verified, dependencies among the descriptors might be discovered by applying Cronbach's α test [37].

Another meta-analysis paper is that of Blangiardo and Richardson [38]. They also scored 2-by-2 contingency tables derived from ranked lists, seeking a "...parsimonious list associated with the strongest evidence of dependence between experiments." Their pioneering work differs from ours three respects.

First is their use of a given number (101) of bins so that a bin could contain all of a subset of descriptors with close p-values. Second, the hypergeometric distribution is the score of the paired bins as shortlists. (By definition, hypergeometric distribution is the chance probability of exactly a given intersection size of subsets of p and q elements from a universe of n elements; RFET is the probability of that number of intersection elements or more, limited by max {min {p, q}}. Thus RFET is a decreasing sum of a finite number of hypergeometric terms, the first of which was the score used by Blangiardo and Richardson.)

Third, and perhaps most importantly, they only scored shortlists of equal length, hence the diagonal of the discrete space of all combinations of bin sizes. By contrast we consider all "rectangular" combinations of shortlists lengths that lie within a larger "square" (such as 20-by-20) of combinations.

The significance of the restriction of consideration to shortlists of equal length can be illustrated as follows. Suppose that two experiments test 100 descriptors for case and control informativeness, providing two ranked lists. Suppose the first experiment is very accurate but the second is not; perhaps the second employs a noisier technology but still might provide a degree of confirmation. Suppose the top four descriptors in the first experiment appear in rank positions 5, 10, 15, 20 of the second, and that no other descriptors in the top twenties are shared. BLANKET correctly selects the top 4 of the first list and the top 20 of the second, with RFET = .00124 < .00424 in Table 1. However, requiring shortlists of equal length forces consideration of the top 5, 10, 15, and 20 of both lists. This results in RFET and hypergeometric distribution p-values all above .2. That is, restricting consideration to shortlists of equal length would find no informative shortlists and in particular would miss the combination (4, 20) found by BLANKET.

Regarding other related papers, we note that Hess and Iyer reported that Fisher's combined p method applied to microarray data from spike-in experiments with RT-qPCR validation usually compared favorably to other methods [4]. As they further noted, current methods for analyzing microarray data do better at ranking genes rather than maintaining stated false positive rates.

Breitling et al., devised the "rank product" method which in simplest form uses multiplication across N experiments of the reciprocal of rank positions of N descriptors, leading to a kind of global ranking [39,40]. In some cases, two logically distinct lines of experimentation might lead to two classes, each including many experiments. The rank product approach might be applied to experiments from one class and then the other, and the two resulting global ranked lists be submitted to BLANKET. For example, in the context of a given case versus control study, a global ranked list could be derived from many microarray experiments. Then the same genes could be ranked from keyword studies of research papers associating them with case outcomes, again producing a global ranked list. Finally, the two global ranked lists, from very different lines of investigation, could be analyzed by BLANKET to discover collectively informative subsets of genes.

An enhancement of BLANKET in gene expression analysis of microarrays might include consistency of fold change. That is, the researcher might require that the genes in the intersection of shortlists all have fold change > 1 or all have fold change < 1 for case versus control. Doing this for randomly generated ranked lists and random fold changes would result in a table like Table 1 but with increased values.

## Conclusions

The BLANKET method provides a visual representation of optimal selections of subsets of informative descriptors. A key observation in our real data is that there can be an abruptly lower (better) RFET score value, going from a plateau of almost 1 to a valley floor of almost 0 values as shortlist lengths are slightly incremented. Furthermore, if upper limits on the shortlist lengths are specified as 10 or 20, then our simulations provide values for RFET scores that allow rejection of the null hypothesis with 95% certainty. In such circumstances, BLANKET can suggest a sharp distinction between slightly too few and slightly too many descriptors, that is, a classifier based upon optimal collective informativeness.

## Authors' contributions

All three wrote sections of the paper. CDJ conceived an initial version of the blanket algorithm; WOW executed early applications and R code and contributed much of the text; DOP contributed refinements regarding applications; and FAW contributed the random permutation design and simulations, R code, and theoretical foundations and analysis.

## Supplementary Material

Additional file 1 **R program for BLANKET**. R program for BLANKET. This program yields a value that can be tested in Table 1 for statistical significance of the discovered shortlists. Click here for file
